# The second report of a new hypomyelinating disease due to a defect in the *VPS11* gene discloses a massive lysosomal involvement

**DOI:** 10.1007/s10545-016-9961-x

**Published:** 2016-07-29

**Authors:** Konstanze Hörtnagel, Inge Krägeloh-Mann, Antje Bornemann, Miriam Döcker, Saskia Biskup, Heidi Mayrhofer, Florian Battke, Gabriele du Bois, Klaus Harzer

**Affiliations:** 1 Praxis für Humangenetik, Paul-Ehrlich-Str. 23, 72076 Tübingen, Germany; 2 CeGaT GmbH, Paul-Ehrlich-Str. 23, 72076 Tübingen, Germany; 3 Department of Neuropediatrics and Neurometabolic Laboratory, Children’s Hospital of the University of Tübingen, Hoppe-Seyler-Str. 1, 72076 Tübingen, Germany; 4 Department of Pathology, Division of Neuropathology, University of Tübingen, Calwer Str. 3, 72076 Tübingen, Germany; 5 Hertie Institute of Clinical Brain Research, University of Tübingen, Otfried-Müller-Str. 27, 72076 Tübingen, Germany; 6 ‘genetikum Stuttgart’, Genetic Counselling and Diagnostics, Lautenschlagerstr. 23, 70173 Stuttgart, Germany

## Abstract

Vesicular protein sorting-associated proteins (VPS, including VPS11) are indispensable in the endocytic network, in particular the endosome-lysosome biogenesis. Exome sequencing revealed the homozygous variant p.Leu387_ Gly395del in the *VPS11* gene in two siblings. On immunoblotting, the mutant VPS11 protein showed a distinctly reduced immunostaining intensity. The children presented with primary and severe developmental delay associated with myoclonic seizures, spastic tetraplegia, trunk and neck hypotonia, blindness, hearing loss, and microcephaly. Neuro-imaging showed severe hypomyelination affecting cerebral and cerebellar white matter and corpus callosum, in the absence of a peripheral neuropathy. Electron microscopy of a skin biopsy revealed clusters of membranous cytoplasmic bodies in dermal unmyelinated nerve axons, and numbers of vacuoles in eccrine sweat glands, similar to what is seen in a classic lysosomal storage disease (LSD). Bone marrow cytology showed a high number of storage macrophages with a micro-vacuolated cytoplasm. Biochemically, changes in urinary glycosphingolipids were reminiscent of those in prosaposin deficiency (another LSD). The clinical and neuro-imaged features in our patients were almost identical to those in some recently reported patients with another variant in the *VPS11* gene, p.Cys846Gly; underlining the presumed pathogenic potential of *VPS11* defects. A new feature was the morphological evidence for lysosomal storage in VPS11 deficiency: This newly characterised disease can be viewed as belonging to the complex field of LSD.

**Electronic supplementary material:**

The online version of this article (doi:10.1007/s10545-016-9961-x) contains supplementary material, which is available to authorized users.

## Introduction

Many gene defects lead to metabolic errors with corresponding clinical symptoms already in early life. In child neurology, the situation is well known that in a patient the neuro-developmental, neuro-imaged and other findings highly suggest the presence of a neurometabolic disease, but classical biochemical, genetic and other laboratory methods have failed to sufficiently contribute to the exact diagnosis of such a progressive disease. In such cases the recent facilities to screen high proportions of the whole exome at once offer a realistic chance (Sawyer et al 2016) to make a genetic diagnosis.

The *VPS11* gene codes for the human homolog of the yeast class C VPS11 protein; about 40 VPS proteins being known for yeast (Kim et al 2001). VPS means Vacuolar (or Vesicle-mediated) Protein Sorting, but the abbreviation is regularly used for the so-called vacuolar protein sorting-associated proteins encoded by *VPS* genes. The mammalian homologs of yeast class C VPS are remarkable for their association with late endosomes and lysosomes (Kim et al 2001). The functions of VPS are not fully understood, but some VPS seem to be implicated in human diseases [for example, chronic apical periodontitis, and genetically caused forms of Parkinson disease (Tang et al 2015)]. There is consent that VPS may be involved in the trafficking of vesicle-enveloped molecules to late endosomes and lysosomes. In this process, parts of the endosomal and early lysosomal membranes seem to fuse with each other; thereby controlling protein sorting and other fundamental processes (Balderhaar and Ungermann 2013). The integration of VPS in the whole endosomal-lysosomal, “endocytic” network, which requires a number of gene products in addition to VPS for its function (Balderhaar and Ungermann 2013), was reviewed in Edvardson et al (2015).

We report on the clinical, morphological, biochemical and genetic findings in two siblings with severe hypomyelination attributed to VPS11 deficiency (V11D). Recently, Edvardson et al (2015) described a mutation in the *VPS11* gene in a group of hitherto undiagnosed patients characterised by a severe neurodegenerative, in particular hypomyelinating disease. Whole exome sequencing (Biskup 2010; Sawyer et al 2016) in our patients revealed another *VPS11* mutation, thus providing the second observation of such a defect in humans. As novel features in V11D, we describe distinct lysosomal storage phenomena, and urinary lipid changes suggestive of lysosomal dysfunction. This shed some light on the possible patho-mechanisms underlying the V11D condition, which seems to represent a novel type of lysosomal storage disease (LSD).

## Clinical report

**Fig. 1:**
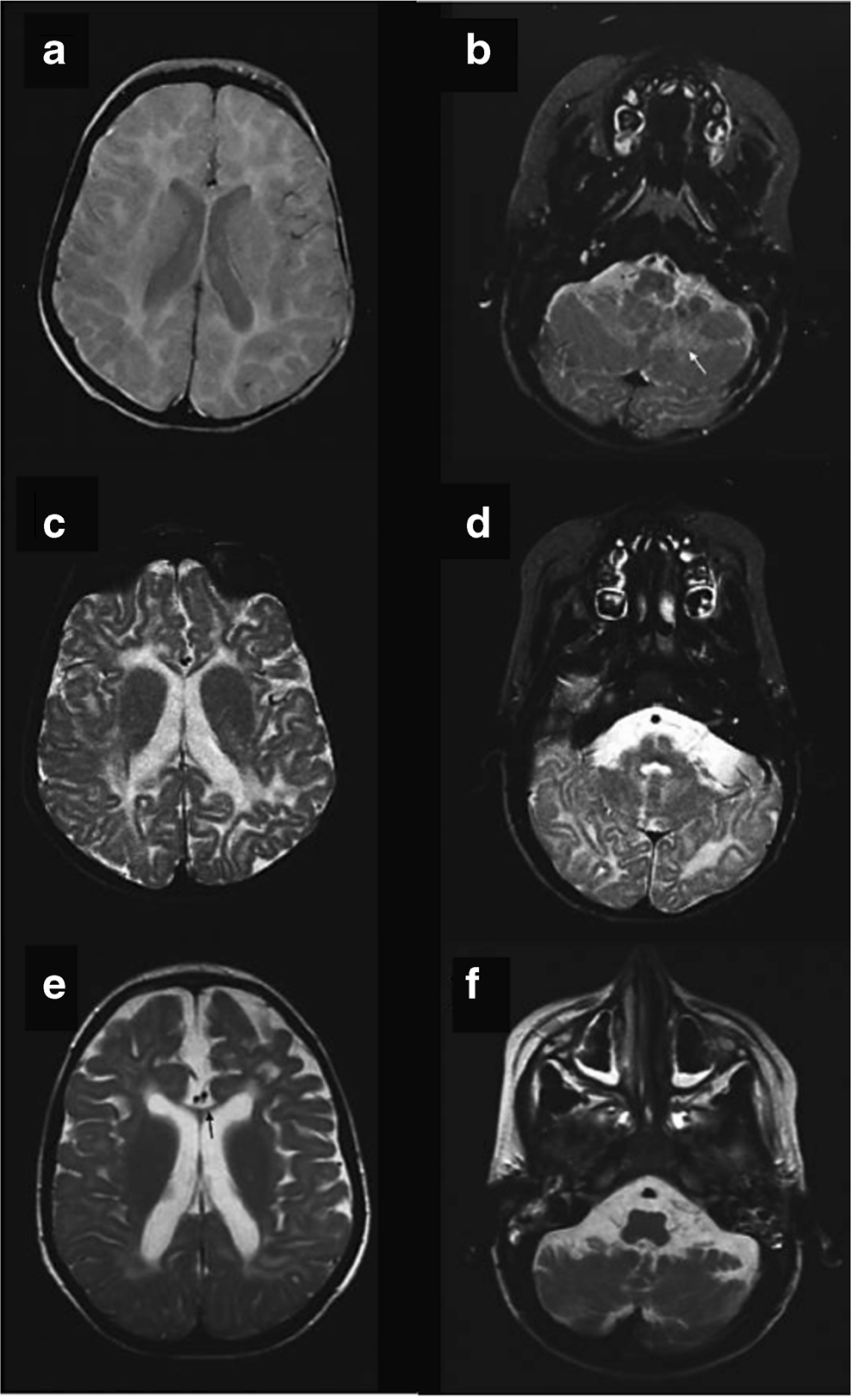
MR imaging of the brain in patient 1 at the age of 12 (**a**,**b**), 27 (**c**,**d**) and 73 months (**e**,**f**). Axial images at the level of the basal ganglia (**a**,**c**,**e**) as well as the cerebellum (**b**,**d**,**f**) show signal changes which indicate hypomyelination: there is high signal on the proton density (**a**) and T2w (**c**,**e**) images of the supratentorial white matter. Inner and outer CSF spaces were mildly enlarged on follow-up and corpus callosum got thin (*black arrow* in **e**) corresponding to atrophy. The cerebellum was not specifically affected by atrophy, but cerebellar white matter was also hypomyelinated, best seen at age 12 months (**b**), *white arrow*

Patient 1 (Pat1, male) was born at 37 weeks of gestation to healthy, consanguineous parents (cousins but with generation skipping). Developmental delay was first recognised at the age of 3 months. At the age of 6 months, fixation and following with the eyes, which were initially acquired, was lost; the boy had a pendular nystagmus and truncal hypotonia, and the head circumference was below the 3rd percentile (P3). Inspiratory stridor was noted. At the age of 12 months, weight and length also were below P3. Opisthotonic posturing, limb hypertonicity and fisting of the hands was reported, and head control was markedly impaired. Liver, spleen and palpable lymph nodes were not enlarged. During his stay at the hospital, short epileptic seizures of variable phenomenology were recognised including sudden extension of the arms, upward gaze of the eyes, myoclonic fits involving the face and shoulders. Laboratory testing revealed slightly elevated liver parameters, and mildly elevated serum lactate (3.8 mmol/l; normal ≤ 2.44). At the age of 27 months, sensorineural hearing loss was diagnosed and hearing aids introduced. At age 34 months, his inspiratory stridor depended on certain body positions, feeding and swallowing had become difficult. He showed simple communicative skills, responded to loud speech and body contact by smiling and turning of the head. Neurologic examination showed spastic tetraplegia with trunk and neck hypotonia; the Babinski sign was positive. Electroencephalograms with polymorphous changes and multifocal spikes had worsened compared to earlier exams. At the age of 54 months, the mother reported on weakness and fatigue, and long lasting common infections. The body measures were all distinctly below P3 but paralleled those of normal growth curves. Neurological examination unchangedly showed the severe tetraspastic disorder with hyperactive tendon reflexes and positive Babinski signs, and almost no gross motor function, and the blindness. Seizures were frequent with twitching of the face and mouth, and jerks of one body half especially when waking up. Occasionally, he experienced grand-mal seizures. Electroencephalograms showed signs of hypsarrhythmia when he was asleep, and multifocal activities when awake. He showed luxation of the left hip, scoliosis and thoracal kyphosis. At the age of 94 months, his seizures worsened, he showed clusters of myoclonic seizures lasting up to 15 min which occurred immediately after waking up. He appeared very weak and hypotonic. Electroencephalograms displayed multifocal sharp-waves and sharp-slow-waves complexes. The size of the liver had increased on sonography (11.3 cm, anterior axillar line) and was now at the upper limit for age. The further course was characterised by almost total immobility, seizures and recurrent lung infections, complications due to a gastro-oesophageal reflux disease and need of gastrostomy. The patient died at 16 years of age. *Cerebrospinal fluid* (CSF) findings including total protein content were normal (at 12 months). Sensory and motor *nerve conduction velocities* (NCV) were unremarkable (at 12, 73 and 94 months). *Ophthalmological examination* at the age of 3 and 8 years revealed normal pupillar reactions, but poor fixation and following of the eyes to lights. The fundi displayed slightly granulated maculae without physiologic reflexes; the optic disks were distinctly pale, interpreted as atrophy of optic nerves corresponding to the observed visual loss. *MR imaging of the brain* at the age of 12, 27 and 73 months years revealed white matter signal changes indicative of hypomyelination (high on T2w and iso- to slightly hyperintense on T1w) of the entire supratentorial white matter, and also the cerebellar white matter (Fig. 1). Inner and outer CSF spaces were mildly enlarged though some progression could be noted, and the corpus callosum got thin (less than 1 mm). MR-spectroscopy of the occipital white matter at the age of 12 months showed reduced N-acetylaspartate, but no evidence for elevated lactate.

Patient 2 (Pat2, female) was 5.5 years younger than her brother (Pat1) and had a similarly severe disease. She deceased at home already at the age of 16 months for unknown reasons. The developmental delay and sequence of clinical features were very similar to her brother’s first 16 months. At the age of 3 months, the mother noted that the baby’s movements were slow and sparse, she hardly turned her head to look at the mother. At the age of 4.5 months, the child’s developmental delay, muscle hypotonia, impaired spontaneous moving and inability to fix with the eyes were documented for the first time. At age 6 months, she had a severe developmental delay with general hypotonia, and almost no spontaneous movements. A spastic movement disorder was suspected, and she exhibited an inspiratory stridor. At the age of 9 months, the mother reported on opisthotonic posturing and little motor activities. At examination, the stridor was marked. She had also cushion-like dorsal swellings on hands and feet, and showed a uvula bipartita. She showed truncal hypotonia and hypertonicity of the extremities, her head control was poor and she showed little movements. Tendon reflexes and Babinski sign were absent. She did not react to light nor followed with the eyes. Electroencephalograms displayed multifocal sharp and spike waves. NCV measuring was normal. Laboratory tests revealed mildly elevated serum lactate and carnitine. Transaminases were at the upper limit of normal, and lactatdehydrogenase distinctly high. On palpation, the liver was 2 cm below the ribs, but, sonographically, the organ was unremarkable. The ophthalmologist found pale optic disks indicating atrophic optic nerves. The child was considered essentially blind. On three attempts, cerebral MR imaging could not be performed because of respiratory and circulatory problems.

## Materials and methods

### DNA samples

Genomic DNA was isolated from fibroblasts of the two patients and from blood samples of the parents using the QIAamp DNA Mini Kit (no. 51306, Qiagen NV, Hilden, Germany) following the manufacturer’s instructions.

### Urinary lipid samples

The 24 h-urines were collected, homogenised by shaking, divided into 10 ml portions and immediately frozen at −20 °C. Thawed portions were immediately lipid-extracted by three subsequent phase partitions of chloroform/methanol/urine or /water mixtures, and the final organic phase was the lipid extract to be used (details on request). Concentrated extracts corresponding to about 10–15 ml urine, and an earlier prepared sulfatide lipid standard (3 to 5 μg) were used for two-dimensional thin-layer chromatography (TLC) on 20 × 20 cm silicagel TLC plates (no. 1.05721, Merck, Darmstadt, Germany) as described (Schlote et al 1991; Paton et al 1992; Kuchař et al 2009). The anisaldehyde/sulfuric lipid staining reagent was used as described (Paton et al 1992). A semi-quantitative method for the determinaton of single glycosphingolipids is described in the Supplementary materials available to this article.

### Exome analysis

The exomes of patient 2 and her parents were sequenced using the following protocol: DNA was enriched using the SureSelectXT V5 exome kit (Agilent, Boeblingen, Germany) according to the manufacturer’s instructions. Sequencing was performed on a HiSeq 2500 sequencer (Illumina, San Diego, CA, USA). On average, 217 million paired reads with a length of 100 bp were produced per exome (220, 220, and 210 million for the index and the two parents, respectively). Reads were demultiplexed with Casava (1.8.2; Illumina) and adapter sequences removed with Skewer (0.1.116). Trimmed reads were mapped with BWA [0.7.2. (Li and Durbin 2009)] using the mem algorithm against the human reference genome (hg19 from UCSC). This yielded an average coverage of 167 for the enriched target regions (117, 198 and 187 for the index and the two parents, respectively). Variant calling, including small insertions and deletions as well as single nucleotide variants (SNVs), was performed using VarScan 2.3 and samtools mpileup 0.1.18 with bcftools (0.1.17) and vcfutils.pl. Calls also found in dbSNP (Database of Single-Nucleotide Polymorphisms, Bethesda, MD, USA), National Library of Medicine (Build ID: 138; http://www.ncbi.nlm.nih.gov/SNP/) or the Exome Variant Server database (NHLBI GO Exome Sequencing Project [ESP], Seattle, WA, USA; http://evs.gs.washington.edu/EVS/) with an allele frequency >5 % were removed. In addition, frequently observed variants in an in-house database produced from the same sequencing technology and enrichment kit were removed (>80 %, minimum number of references: 21). Transcript and protein alterations (functional consequences) were annotated with in-house software (unpublished) using a proprietary transcript database based on ENSEMBL (v75), RefSeq (as mapped in UCSC on 2014-11-06), and CCDS (release 15). Only variants potentially changing the protein sequence were used for further analysis; intronic, UTR and synonymous mutations were removed. Finally, the variant list was filtered for all variants occurring in both affected family members using a self-developed tool (unpublished). A trio analysis was conducted to further filter the variant list. The remaining SNVs and INDELs of the index patient were used to check if, (i) the index patient is compound heterozygous and each parent carries one heterozygous mutation, (ii) index patient has a homozygous mutation and parents are heterozygous, and, (iii) index patient has a de-novo mutation. All three cases were automatically computed by a self-developed tool (unpublished). Sanger sequencing for VPS11 analysis of patient 1 and validation of NGS results was carried out using standard PCR-based techniques and BigDye (Life Technologies GmbH, Darmstadt, Germany) chemistry (primers available on request).

### Immunoblot preparation

Fibroblasts were lysed in PBS/1 % Triton X-100 and centrifuged for supernatants. Lysates with 30 μg protein were separated using sodium dodecyl sulfate polyacrylamide gel electrophoresis (8 % polyacrylamide; protein ladder NEB P7711) and transferred to PVDF transfer membranes (no. IPVH00010, Immobilon-P Millipore, Merck, Darmstadt, Germany). These membranes were then probed with antibodies overnight at 4 °C against VPS11 (no. 19140-1-AP, Proteintech, Manchester, U.K.), Jak2 (no. 3229, Cell Signaling, Bio-Rad, München, Germany) and tubulin (no. ab6160, Abcam, Cambridge, U.K.), respectively, washed in TBS and incubated for 1 h with secondary antibodies conjugated with peroxidase (no. 7074, Cell Signaling, and no. 6845-1, Abcam, respectively). The membranes were developed using Amersham ECL-Prime Western Blotting Detection Reagent (no. RPN2232, GE Healthcare, Merck) according to the manufacturers instructions and exposed to an X-ray film. Quantitation was performed using a densitometer.

## Results and discussion

### The VPS11 mutation in relation to the clinical picture

**Fig. 2:**
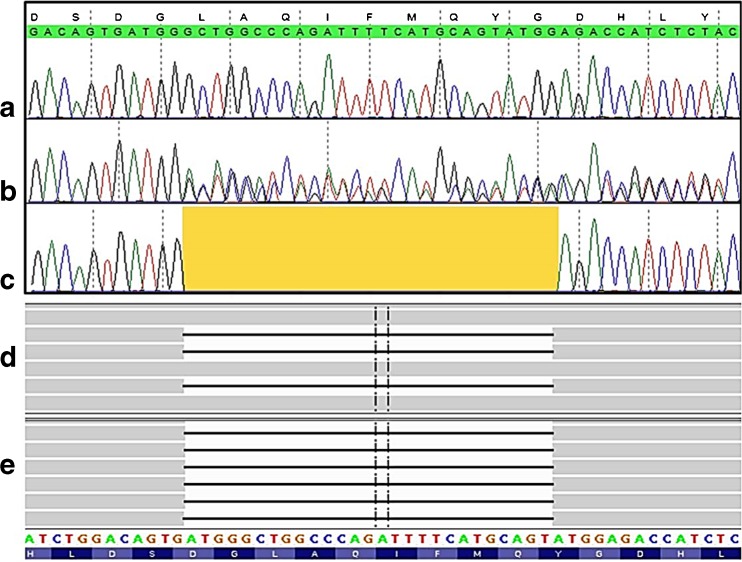
Sequencing data of the *VPS11* mutation, i.e. the deletion given as the *yellow area* in **c** or, in **d** and **e**, the *horizontal black lines*. Shown are Sanger sequences of **a** wild type, **b** mother (heterozygous), **c** patient 2 (homozygous), and reads obtained from the exome next generation sequencing from **d** mother (heterozygous) and **e** patient 2 (homozygous). The apparent shift in position of the deletion in Sanger vs. NGS sequencing reads is due to the identical ATGG characterising in wild type both sides of the region that is deleted in **b** to **e**. The calling algorithm used the most 5′ position in contrast to the HGVS nomenclature in which the most 3′ position is recommended

While preparing the description of these patients with the new defect in the *VPS11* gene, the publication by Edvardson et al (2015) appeared that described eight patients in four Ashkenazi-Jewish families with a defect in the *VPS11* gene identified as p.Cys846Gly. The *VPS11* variant in our patients, p.Leu387_Gly395del (c.1158_1184del, NM_021729.5) (Fig. 2), is a hitherto unknown in frame deletion of nine amino acids in a highly conserved region of the *VPS11* gene (deletion not observed in public databases). The deletion variant was homozygous in our patients and heterozygous in both parents. In Fig. 2, the wild type sequence scales at the top (Sanger sequencing) and the bottom (NGS) are not congruent for the reasons given in the figure legend. In Fig. 2, lane b, only one of the heterozygous sequences is identified by the wild type scale at the top. The other sequence is shifted due to the deletion (peak colours/identities deviating from the wild type sequence in a). Interestingly, the clinical picture described in the patients bearing the p.Cys846Gly was very similar to that observed in our patients. The ‘appendicular spasticity’ (Edvardson et al 2015) which means the spastic peripheral limb muscles as a hypertonic system appending to the hypotonic system of the remaining body, essentially the trunk, was a leading symptom shared with our patients. Moreover, the acquired microcephaly, seizure manifestations and severe hypomyelination affecting cerebral and cerebellar white matter with thinning of the corpus callosum (Fig. 1), were also convincingly shared. This clinical similarity of the described (Jewish) patients (Edvardson et al 2015) and our (non-Jewish, Turkish) patients suggested that the *VPS11* defects were causative for the disease, in accordance to the absence of any other reported severe defect in the whole exome analyses. Our second observation of a *VPS11* defect has now strongly supported the pathogenic impact of such a defect, which was anticipated by Edvardson et al (2015). The different mutations in the same gene leading to the same disease in both observations reflect a typical situation in many genetic disorders.

### The protein phenotype in VPS11 deficiency

**Fig. 3:**
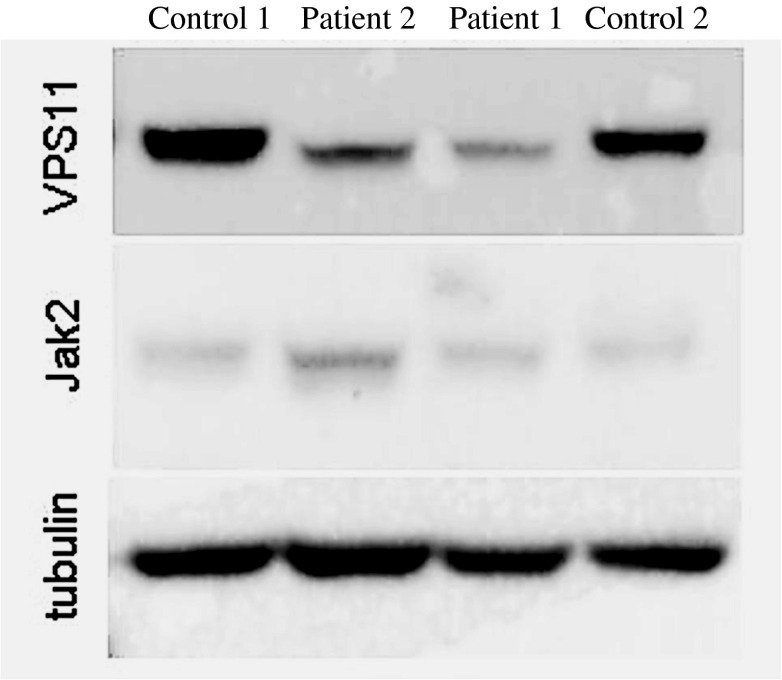
Immunoblots of normal and VPS11-mutated fibroblast extracts (each with 30 μg protein). It is seen that in patients 1 and 2 the VPS11 bands have reduced immunostaining intensities as compared to the two normal controls. Normalisation of the quantified VPS11 stain intensities on the basis of the Jak2 (5 μg per band) intensities revealed for the VPS11/Jak2 ratios, 0.26 and 0.31 in patient 2 and 1, respectively, as compared to 1.63 and 1.58 in the controls. Similar normalisation for the VPS11/tubulin (5 μg) ratios revealed 0.2 and 0.134 in patient 2 and 1, respectively, as compared to 0.72 and 0.47 in the controls. For a possibly slightly enhanced migration rate of the mutant VPS11, see “Results and discussion”

The immunoblot shown in Fig. 3 compares the mutated protein in our patients to that in normal controls. Patients 1 and 2 have less intensely immunostained VPS 11 bands as compared to the controls. Normalisation of the staining intensities on the basis of reference proteins revealed the distinct differences given in the legend to Fig. 3. However, the distinct reduction of the amount of the mutant VPS11 cannot, yet, be explained. A deletion-mediated instability of the transcript, or a rapid decay of the mutant VPS11 protein initiated by the cellular control systems might have been responsible. Although in Fig. 3 the mutant VPS11 bands apparently had slightly faster migration rates as compared to the controls, this difference may be questioned because the Jak2 standard in the patient 2 lane behaved similarly (by an artifact due to the overloaded Jak2 band?). However, in the patient 1 lane, the Jak2 standard did obviously not behave atypically. So, the migration rate of the mutant VPS11 might still have been slightly increased due to the reduction in size, as compared to normal VPS11, by the described nine amino acid-deletion.

### The lysosomal involvement in VPS11 deficiency

**Fig. 4:**
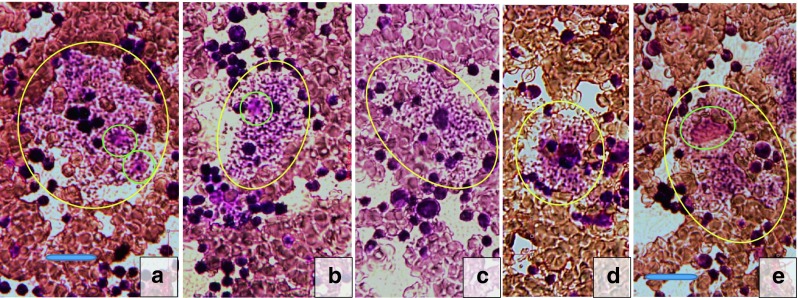
Bone marrow cytology in present patient 1 (male). Pathologic cells *encircled in yellow. Horizontal blue bars* in graphs mark 20 μm. Parts **a** to **e**, storage macrophages filled with granules which on direct microscopy mostly appear as micro-vesicles. The reddish nuclei in parts **a** and **b** are *encircled in green*, and two of them display distinct dark nucleoli. In parts **c** and **d**, the nuclei are hidden by engulfed or overlaid leukocytic cells. In part **e**, a relative large reddish nucleus *encircled in green* is loosely associated with a granular and micro-vacuolar mass which does not seem to be coated by a cell membrane (disintegrating storage macrophage?)

**Fig. 5:**
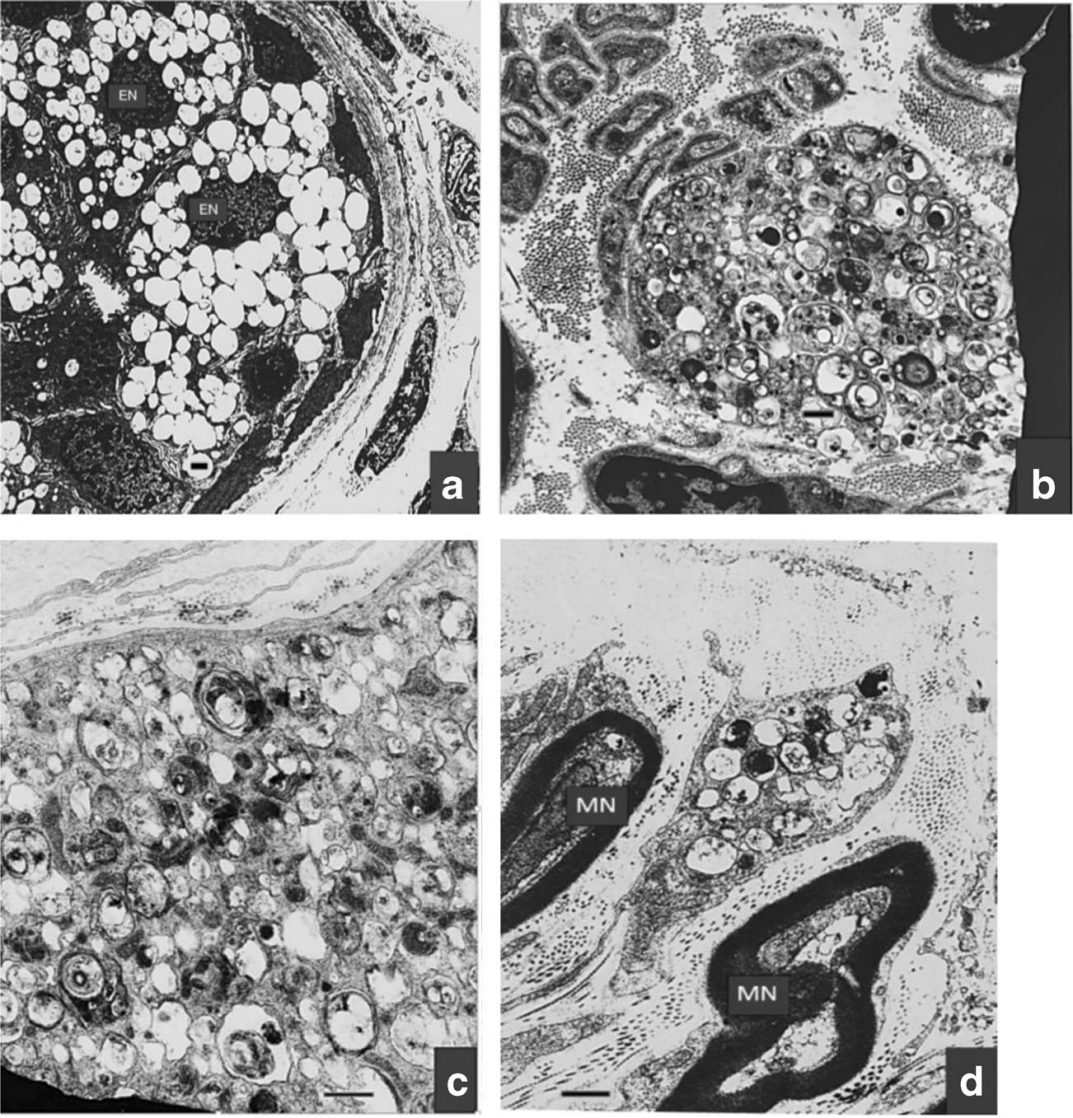
Electron microscopy of skin biopsies from the present patients. *Horizontal bars* mark 1 μm in each graph. **a**, eccrine sweat gland in patient 1 (*EN*, nuclei of gland epithelia); note the numberless, almost clear vacuoles representing storage lysosomes. **b**, large round area in the mid: unmyelinated nerve ending axon filled with sometimes dark, polymorphous membranous cytoplasmic bodies representing lipid and other materials storing lysosomes, in patient 1. **c**, this finding in patient 2 is identical to that shown in **b. d**, also from patient 2, shows in the mid a process of a fibrocyte containing storage bodies similar to those seen in **b** and **c** (*MN*, normally myelinated dermal nerve fibres)

Some contribution to possible disease patho-mechanisms in our patients came from “nature experiments”. The natural course of their disease resulted in a highly pathologic involvement of the lysosomal compartments in different cell types. The morphologic changes (Figs. 4 and 5) left no doubt that a leading aspect of the disease (now called VPS11 deficiency [V11D]) was that of a lysosomal storage disease (LSD). The storage macrophages seen in the bone marrow smear from patient 1 (Fig. 4) resembled the storage cells in infantile sphingomyelinase deficiency (Niemann-Pick disease type A), although, in contrast to the Niemann-Pick A cells, they seemed to be mechanically instable, as could be concluded from the occurrence of high numbers of cell debris with the appearance of irregular clusters of storage vacuoles unrelated to any cell nuclei. Unexplained were the very distinct, condensed nucleoli in the storage cell nuclei (Fig. 4; part a, lower green encircled nucleus; part b, green encircled nucleus). Electron microscopy of the skin biopsies of both patients revealed numbers of lysosome-equivalent clear vacuoles in the eccrine sweat glands (Fig. 5a), but also areas with mostly dense, multiform membranous cytoplasmic bodies (Fig. 5b,c) located to unmyelinated axons, as well as less dense bodies in fibrocyte processes (Fig. 5d). The structures in Fig. 5b,c were identical to the structures shown, e.g., in the LSD type known as sialidosis (Martin et al 1981). Figure 5a and b belonged to patient 1, Fig. 5c and d to patient 2; the depicted ultrastructures were identical. The frequency and intensity of these ultrastructural storage phenomena were much higher in patient 1 (skin biopsy taken at age 73 months) than in patient 2 (biopsy at 7 months). This aspect of lysosomal involvement in V11D was not reported to be characteristic for the patients investigated by Edvardson et al (2015), although the authors spoke of mild accumulation of cargo in the late endosome. The late endosome is close to the lysosome and accumulates substances in some LSD. The apparent difference in lysosomal involvement between the observation by Edvardson et al (2015) and ours could be due to the different nature of the *VSP11* mutations in both: p.Cys846Gly and p.Leu387_Gly395del affect very different though conserved regions of the gene. The different mutational changes in the VPS11 proteins may trigger different tissue phenotypes, while they lead to almost identical clinical phenotypes of the disease. Whether the lysosomal involvement shown in Figs. 4 and 5 in fact is an expression also of the pathogenic mechanisms which lead to the patients’ brain disease, is unclear. However, by analogy to many known LSD, such an interpretation seems to be admissible. If correct, this interpretation should predict that the patients described by Edvardson et al (2015), when studied in the same way as our patients, would show a similar lysosomal involvement which, only in quantitative terms, might be less distinct than in our patients.

### A biochemical abnormality in VPS11 deficiency

**Fig. 6:**
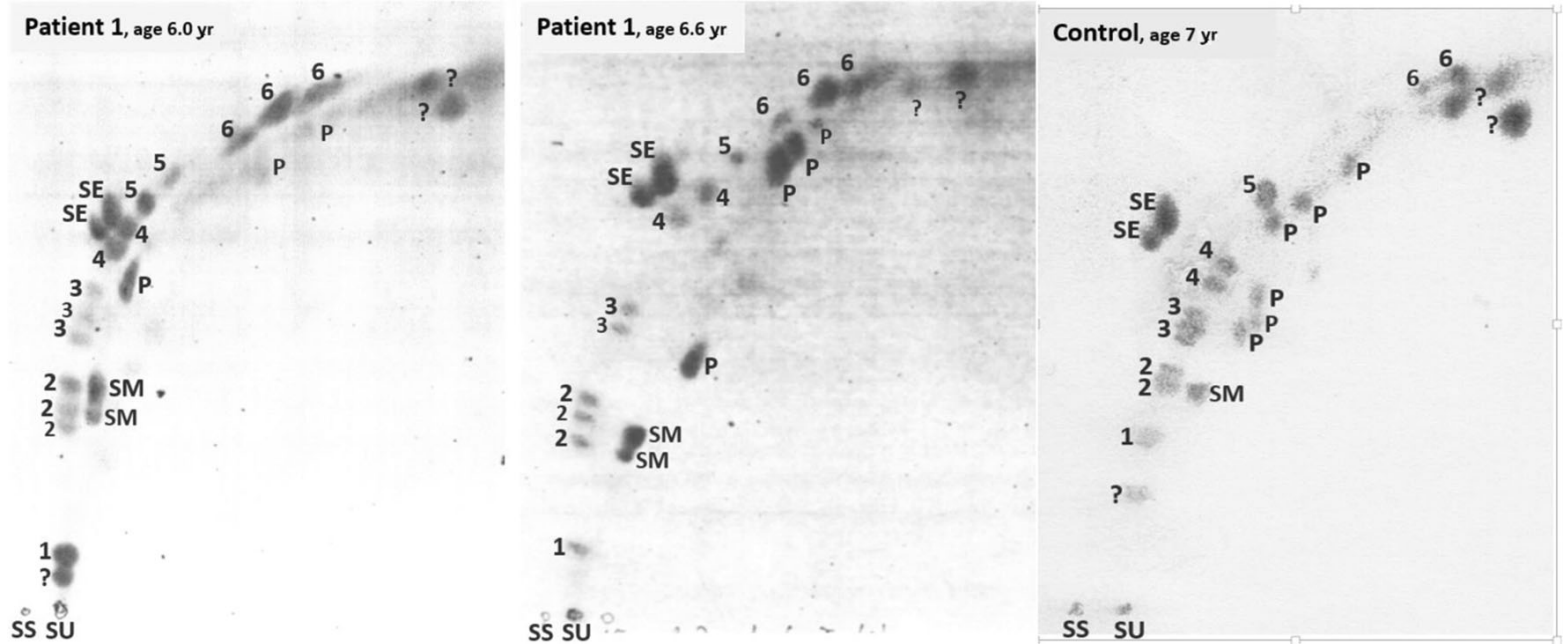
Urinary lipids in two independent samples from patient 1, and a selected normal control (see Supplementary materials available to this article). On the stained, two-dimensional thin-layer chromatograms, the following glycosphingolipid groups were identified: *1*, GM_3_ ganglioside; *2*, tetrahexosylceramides; *3*, trihexosylceramides; *4*, sulfatides; *5*, dihexosylceramides; *6*, monohexosylceramides. Morover, some phospholipids were identified and given the symbol *P* or, in the case of sphingomyelin, *SM*. The *?* denotes unidentified compounds. *SU* is the start point for the urinary total lipid extract and *SS* that for the external standard (sulfatides, 3 to 5 μg); *SE* is this standard which ran slightly higher than the patients spots no. 4 due to its different fatty-acid composition. Note the more intensely stained spots no. 6, 4 and 2 and additional 6 and 2 spots in *Patient 1* as compared to *Control*, viewed as specific glycosphingolipid elevations in the patient. For spots no. 1, see Supplementary materials, in which also a semi-quantitative evaluation of the spot staining intensities as measures of lipid concentrations is described

Some types of glycosphingolipids are known to have elevated urinary levels in lysosomal lipidoses such as metachromatic leukodystrophy, Fabry disease and prosaposin deficiency (Kuchař et al 2009). Patient 1 was remarkable for the combined elevations of monohexosylceramides, sulfatides, tetrahexosylceramides and other glycosphingolipids in two independent urine samples as compared to an age-matched control (Fig. 6). In the figure, the corresponding lipid spots seen on two-dimensional thin layer chromatograms were increased in their staining intensity as compared to the shown control chromatogram. The glycolipid pattern had some similarity to the pattern seen in prosaposin deficiency which is a severe lysosomal lipidosis (Paton et al 1992; Kuchař et al 2009). The lipid analyses were performed 20 years ago. Patient 1 died a decade ago, and no urinary samples had been preserved for a confirmatory mass spectrometric analysis. However, we had some experience with the identification of glycosphingolipids and the differentiation of normal and lysosomal defect-associated, elevated levels on thin-layer chromatograms (Paton et al 1992). The different fundamental and methodological caveats of this type of urinary lipid analysis as well as a semi-quantitative method for the lipid determination on the present chromatograms are described in the Supplementary materials available to this article. We concluded that the shown urinary glycosphingolipid abnormality in patient 1 is a marker of the lysosomal involvement in V11D.

### The hypomyelination in VPS11 deficiency

A distinct clinical feature of our patients as well as those described by Edvardson et al (2015) is hypomyelination, allowing to include V11D within hypomyelinating diseases (HMD). HMD comprise a group characterised by a permanent, substantial deficit in myelin deposition in the brain (Barkovich 2015). They are genetically but also clinically heterogeneous. The gene defects were proposed to trigger impaired functions of the oligodendrocyte and its cellular precursors (Pouwels et al 2014). Some LSD such as infantile GM_1_ and GM_2_ gangliosidosis, Salla disease and fucosidosis were shown to have neuro-imaging characteristics of HMD, allowing to include them also within HMD (Steenweg et al 2010). Some clinical features in our V11D patients, for example, nystagmus and inspiratory stridor (attributed to the pharyngeal and tongue slackness), were shared with classical HMD, in particular Pelizaeus-Merzbacher disease (PMD). Disease courses with the absence of almost any developmental progress and without a predominant seizure disorder, however, are rare in classical PMD or other genetically defined HMD. The manifestation of some classical LSD, though only in their early onset forms with a severe clinical course, as HMD (Steenweg et al 2010), might suggest that also in V11D lysosomal dysfunctions trigger the hypomyelination. However, the lysosomal affection in V11D might be different from that in classical LSD.

For the new disease, V11D, it can be expected that studies of V11D cells in vitro will provide us with interesting data on the normal and defective lysosome biogenesis, and give insight into the functions of VPS11.

## Conclusions

The role of VPS11 in the complex endosomal-lysosomal pathways might explain that the V11D condition has several phenotypic aspects of a LSD. The morphological evidence for lysosomal storage in V11D came, at the ultrastructural level, from high numbers of membranous cytoplasmic bodies in dermal unmyelinated axons and groups of vacuoles in eccrine sweat glands, and, at the bone marrow cytological level, from a high number of storage macrophages with a foamy cytoplasm. So, classical morphologic approaches should remain additional diagnostic options, as neuro-pathologic findings in biopsies may suggest new disease aspects which might not directly become obvious from the unravelled genotype. In addition to the morphologic evidence, a biochemical marker of a lysosomal dysfunction in the present V11D patients was suggested to exist by the observed urinary glycosphingolipid abnormality resembling the changes in prosaposin deficiency. The defective endo-lysosomal molecule trafficking in V11D might have the consequence that different proteins (for example, prosaposin) destined to function in the mature lysosome could not reach this compartment, leading to a scenario well comparable to that in many LSD with primary defects of lysosomal proteins. According to the novel findings, V11D can be viewed as belonging to LSD which today include many types in which the metabolic bases are very different from the old principle of a deficient lysosomal enzyme leading to lysosomal storage. In accordance with the substantial hypomyelination known for some severe, early-onset forms of classical LSD, and in some correspondence to the here described V11D disease, lysosomal storage processes can be viewed as some of the candidates for the largely puzzling pathogenic factors in HMD.

*CSF* cerebrospinal fluid, *HMD* hypomyelinating disease, *LSD* lysosomal storage disease, *MR* magnetic resonance, *NCV* nerve conduction velocity, *NGS* next generation sequencing, *P3* 3rd percentile, *PMD* Pelizaeus-Merzbacher disease, *TLC* thin layer chromatography, *T2w* T2-weighted image, *V11D* VPS11 deficiency

## Electronic supplementary material

Below is the link to the electronic supplementary material.

ESM 1 (DOCX 1716 kb)

## References

[CR1] Balderhaar HJ, Ungermann C (2013). CORVET and HOPS tethering complexes – coordinators of endosome and lysosome fusion. J Cell Sci.

[CR2] Barkovich J (2015). Hypomyelinating disorders: an MRI approach. Neurobiol Dis.

[CR3] Biskup S (2010). Molekulargenetische und zytogenetische Diagnostik. Hochdurchsatz-Sequenzierung in der Humangenetischen Diagnostik. Next-generation sequencing in genetic diagnostics. Laboratoriumsmedizin.

[CR4] Edvardson S, Gerhard F, Jalas C, Lachmann J, Golan D, Saada A, Shaag A, Ungermann C, Elpeleg O (2015). Hypomyelination and developmental delay associated with VPS11 mutation in Ashkenazi-Jewish patients. J Med Genet.

[CR5] Kim BY, Krämer H, Yamamoto A, Kominami E, Kohsaka S, Akazawa C (2001). Molecular characterization of mammalian homologues of class C Vps proteins that interact with syntaxin-7. J Biol Chem.

[CR6] Kuchař L, Ledvinová J, Hřebíček M, Myšková H, Dvořáková L, Berná L, Chrastina P, Asfaw B, Elleder M, Petermöller M, Mayrhofer H, Staudt M, Krägeloh-Mann I, Paton BC, Harzer K (2009). Prosaposin deficiency and saposin B deficiency (activator-deficient metachromatic leukodystrophy): report on two patients detected by analysis of urinary sphingolipids and carrying novel PSAP gene mutations. Am J Med Genet.

[CR7] Li H, Durbin R (2009). Fast and accurate short read alignment with Burrows-Wheeler transform. Bioinformatics.

[CR8] Martin JJ, Libert J, Ceuterick C, Tettamanti G, Durand P, Di Donato S (1981). Conjunctival and skin biopsies in sialidoses. Perspectives in inherited metabolic diseases.

[CR9] Paton BC, Schmid B, Kustermann-Kuhn B, Poulos A, Harzer K (1992). Additional biochemical findings in a patient and fetal sibling with a genetic defect in the sphingolipid activator protein (SAP) precursor, prosaposin. Evidence for a deficiency in SAP-1 and for a normal lysosomal neuraminidase. Biochem J.

[CR10] Pouwels PJ, Vanderver A, Bernard G, Wolf NI, Dreha-Kulczewksi SF, Deoni SC, Bertini E, Kohlschütter A, Richardson W, Ffrench-Constant C, Köhler W, Rowitch D, Barkovich AJ (2014). Hypomyelinating leukodystrophies: translational research progress and prospects. Ann Neurol.

[CR11] Sawyer SL, Hartley T, Dyment DA, Beaulieu CL, Schwartzentruber J, Smith A, Bedford HM, Bernard G, Bernier FP, Brais B, Bulman DE, Warman Chardon J, Chitayat D, Deladoëy J, Fernandez BA, Frosk P, Geraghty MT, Gerull B, Gibson W, Gow RM, Graham GE, Green JS, Heon E, Horvath G, Innes AM, Jabado N, Kim RH, Koenekoop RK, Khan A, Lehmann OJ, Mendoza-Londono R, Michaud JL, Nikkel SM, Penney LS, Polychronakos C, Richer J, Rouleau GA, Samuels ME, Siu VM, Suchowersky O, Tarnopolsky MA, Yoon G, Zahir FR, Majewski J, Boycott KM, FORGE Canada Consortium; Care4Rare Canada Consortium (2016). Utility of whole-exome sequencing for those near the end of the diagnostic odyssey: time to address gaps in care. Clin Genet.

[CR12] Schlote W, Harzer K, Christomanou H, Paton BC, Kustermann-Kuhn B, Schmid B, Seeger J, Beudt U, Schuster I, Langenbeck U (1991). Sphingolipid activator protein 1 deficiency in metachromatic leucodystrophy with normal arylsulphatase A activity. A clinical, morphological, biochemical, and immunological study. Eur J Pediatr.

[CR13] Steenweg ME, Vanderver A, Blaser S, Bizzi A, de Koning TJ, Mancini GMS, van Wieringen WN, Barkhof F, Wolf NI, van der Knaap MS (2010). Magnetic resonance imaging pattern recognition in hypomyelinating disorders. Brain.

[CR14] Tang FL, Erion JR, Tian Y, Liu W, Yin DM, Ye J, Tang B, Mei L, Xiong WC (2015). VPS35 in dopamine neurons is required for endosome-to-golgi retrieval of Lamp2a, a receptor of chaperone-mediated autophagy that is critical for α-synuclein degradation and prevention of pathogenesis of Parkinson’s disease. J Neurosci.

