# Supplementary material for: The second report of a new hypomyelinating disease due to a defect in the VPS11 gene discloses a massive lysosomal involvement
Source: J Inherit Metab Dis. 2016 Jul 29;39(6):849–57. doi: 10.1007/s10545-016-9961-x (PMC5065605; doi:10.1007/s10545-016-9961-x)
Supplement: Supplementary file 1 — (DOCX 1716 kb) [file 10545_2016_9961_MOESM1_ESM.docx]

Supplementary Materials

Ref.: Main article: *The second report of a new hypomyelinating disease due to a defect in the VPS11 gene discloses a massive lysosomal involvement*

S1: Analysis of urinary glycosphingolipids using two-dimensional thin layer chromatography

S1.1 Introduction

It is important to be aware of the extreme variability in the chemical composition of urine samples. The found concentration of a given metabolite requires to be appropriately normalised for any conclusion drawn from the metabolite’s abundance. A given metabolite concentration in the urinary 24-h-sample – which should be used whenever possible - may be standardised by the found creatinine concentration. However, in the assay of urinary lipids such as cholesterol, phospholipids and glycosphingolipids (CPGL), a standardisation by water-soluble substances including creatinine was not satisfactory. Also no single type of the CPGL themselves seemed to be useful as a reliable standardisation parameter (SP), although sphingomyelin was tried with a limited success (Kuchař et al. 2009). There is general consent that urinary CPGL essentially originate from renal, in particular tubulus cells (RTC), numbers of which are physiologically released into the urine. It was concluded that the number of RTC contained in a given urine sample could well serve as a SP when determining urinary CPGL, while water-soluble substances should not monitor the mass of CPGL which are bound to RTC membranes and separate from the watery pool. However, the number of urinary RTC cannot easily be counted because most of these cells are disrupted. Thus, as a parameter which should essentially parallel the number of RTC and be, therefore, also useful as a SP for single CPGL, the total urinary CPGL concentration was envisaged. However, the here described approach, for methodological reasons, had to reduce the sum of CPGL as a theoretical SP, to the sum of glycosphingolipids and phospholipids as the practically used SP for single lipids. Very hydrophobic compounds such as cholesterol and others could not be regarded. Despite this restriction the obtained results seem to characterise some specific lipid changes in the present patient 1 with VPS11 deficiency (see main article).

S1.2 Methods

The preparation of urinary lipid extracts, two-dimensional lipid thin layer chromatography (TLC) and staining of separated lipids were performed as indicated in the main article. One third of the prepared lipid extracts was applied per chromatogram. The single lipids of interest were identified according to our earlier experience (Schlote et al. 1991; Paton et al. 1992; Kuchař et al. 2009). The chromatograms from patient 1 as well as those from 7 normal controls (age 4 to 8 years) were inspected for the averaged stain intensity of the lipid spots. That control chromatogram whose inspected stain intensity was most similar to the intensity of the patient chromatograms was selected; it was that with the highest stain intensity of all 7 controls. The patient chromatograms and the selected control chromatogram were electronically scanned and the images analysed with an image processing program (Microsoft Word 13). Each distinct individual glycosphingolipid and phospholipid including sphingomyelin spot (n = 17 to 19 per chromatogram; excluding cholesterol and other very hydrophobic compounds which were not sufficiently separated) was gradually extinguished by increasing the imaging brightness according to the program. The percent of brightness increase required until the spot was completely extinguished was recorded and named spot extinction (SE). Calibration curves with lipid standards from 1 to 7µg per spot were almost linear. The mean of the 17 to 19 SE per chromatogram (SEm) was calculated. SEm was essentially proportional to the total mass of lipids studied per chromatogram. The mean of the SEm values of all evaluated chromatograms was taken for the standardisation of the volume of lipid extract to be applied to a repeated series of the chromatograms from the same urinary lipid extracts. Now with the same amount of total studied lipids on each chromatogram (for example, 100 µg), TLC was repeated. The lipid spots were again analysed for their SE values. The SE values from the patient chromatograms were compared to those in the control chromatogram. The increases of SE values in the patient as compared to the control were given the classes, distinctly increased, moderately increased, slightly increased, not increased. For a given lipid spot, the classes were formed according to the ranges of the SE ratios between patient and control (SEpatient/SEcontrol): range >3 (distinctly increased); >2 to 3 (moderately increased); >1.2 to 2 (slightly increased); 0.8 to 1.2 (slightly reduced or increased).

S1.3 Results

The figure repeated from the main article (Fig. 6) shows 3 chromatograms adjusted to the same lipid content in each chromatogram (see section S1.2).


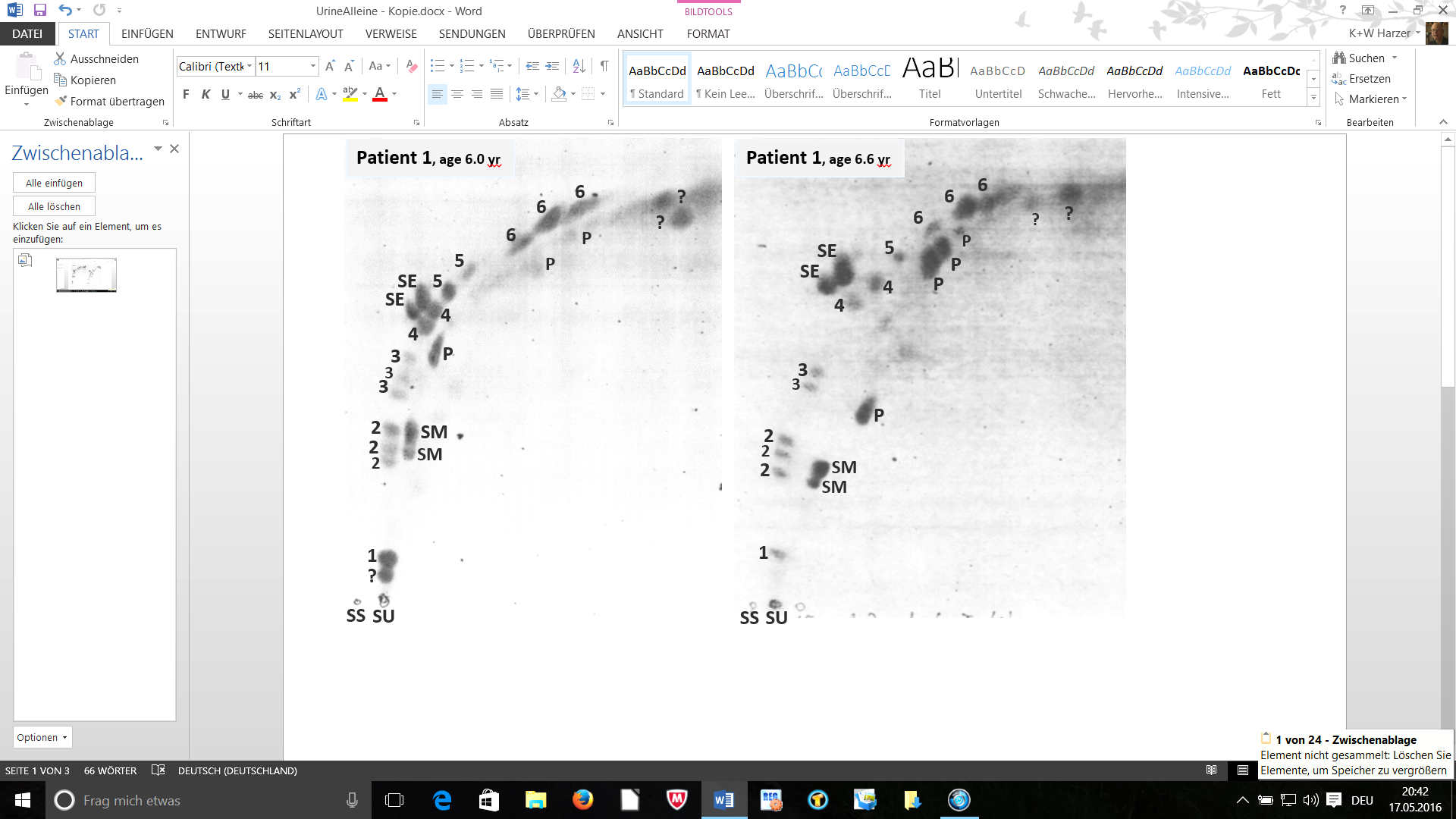

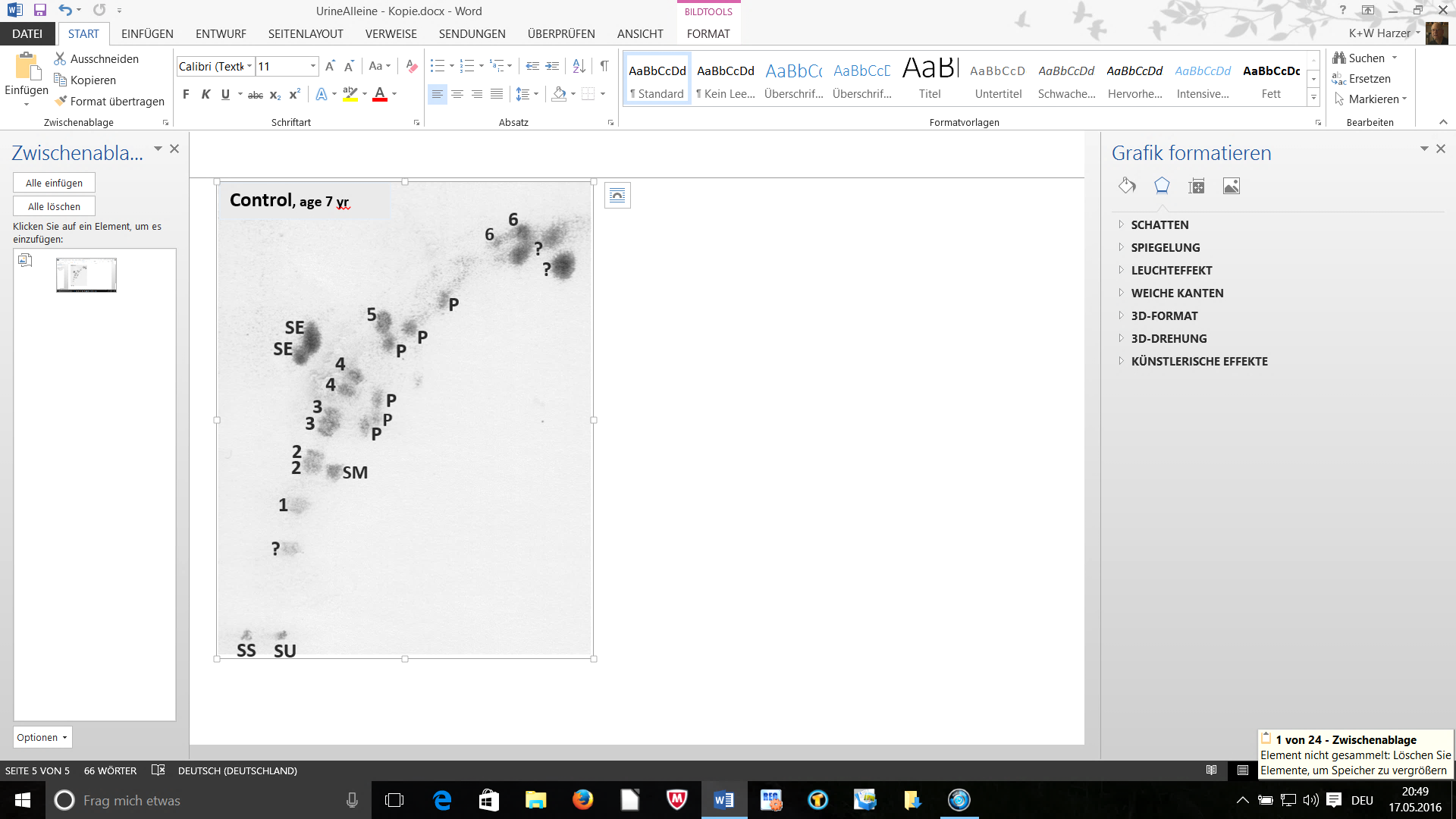


Legend: Glycosphingolipid group numbers: **1**, GM_3_ ganglioside; **2**, tetrahexosylceramides; **3**, trihexosylceramides; **4**, sulfatides; **5**, dihexosylceramides; **6**, monohexosylceramides. Other symbols: **P**, phospholipids except sphingomyelin; **SM**, sphingomyelin. For the remaining symbols, see legend to Fig. 6 in the main article

In patient 1 (left and middle chromatograms, from the 1^st^ and 2^nd^ urinalysis), the sphingolipid 1 (see legend) was distinctly or not increased, sphingolipid group 2 was moderately increased, group 3 was not increased or slightly decreased, group 4 was distinctly or moderately increased, group 5 was slightly increased or slightly decreased, group 6 was distinctly increased. Moreover, the phospholipids P were distinctly or slightly increased, SM was distinctly or moderately increased. The variation between the 1^st^ and 2^nd^ patient urinalysis in the differences against the control is explained in section S1.4.5.

S1.4 Critical remarks

S1.4.1 Method

Although the described lipid estimation method was fundamentally quantitative, the obtained results were reduced to a semi-quantitative scale (distinct, moderate, slight, and no lipid increases in the studied patient) because the variabilities were not completely known, and the methodological concept may not absolutely be free of potential errors. We did not want to simulate more precision of the assays than was realistically achieved.

The use of the simple method, lipid thin layer chromatography (TLC), instead of the modern techniques such as tandem mass spectrometry (TMS), was not viewed as having prevented valid conclusions on the lipid changes in the studied patient. TLC allowed us to directly see certain urinary lipid changes in the context of many other lipids’ abundances, which may help to correctly interpret the changes. Higher precision of a measured individual lipid signal in TMS as compared to TLC will not reduce this context problem.

S1.4.2 Standardisation of individual urinary lipid concentrations

Our approach used the mean intensity of 17 to 19 individual lipid signals as a standardisation parameter (SP), which measure is essentially proportional to the total lipid mass analysed. This SP is not fully representative for the total urinary lipid content which, with many additional lipid compounds included, might lead to slightly different lipid values. This (minor) problem could not be solved. Another problem may be seen in the use of the urinary lipid mass as a SP for single lipids in the case that a given urinary sample is supposed to have some lipid specifically elevated, as in the present patient. Indeed, this may lead to a small under-estimation of pathologically elevated lipids, because the SP calculated for the patient urine was “biased” by the elevated lipids. In other urine samples, similar biases could come from “accidentally” (not specifically) elevated lipids, for example, one or more of the highly variable urinary phospholipids. On the other side, if a normal control urine sample was free of such “accidental biases” of the SP (as, presumably, being the case in the here used control, see figure), the estimated control lipid concentrations would appear too high when compared to negatively biased concentrations which could occur in the patient. At a cellular level, when correlating the lipid amount in the SP with the number of renal cells (RTC) analysed (see section S1.1), the lipid amount in the patient’s SP could simulate a too high number of RTC because the lipid amount per RTC could be increased in the patient; again leading to an under-estimation of the single lipids in the patient.

1.4.3 Two different lipid pools?

We hypothesised that in the case of patient 1 with a lysosomal storage condition (see main article) there are two lipid pools, the one essentially with the accumulated glycosphingolipids sequestered and fixed in the lysosomes of RTC (see section S1.1), and the other essentially with the extra-lysosomal lipids which may undergo their metabolism by a majority “as usually” even in the present lysosomal storage process, and despite their partial dependence also on the degradation within the lysosome. If this hypothesis is correct, the concentrations of the lysosomally accumulated glycosphingolipids in patient 1 should not strongly be paralleled by the concentrations of the essentially extra-lysosomal lipids such as phospholipids including sphingomyelin. Therefore, a common SP for all these lipids as used in this study may bias the estimated lipid concentrations. As to the glycosphingolipid G_M3_ ganglioside (no. 1 in the figure), this polar, “versatile” lipid seems to have very different concentrations under different conditions and in different cell systems where it is sometimes the most abundant ganglioside (Iwamori and Nagai 1978, Tsukuda et al. 2012). Its quantitative involvement in different lysosomal lipid storage processes is also highly variable.

S1.4.4 Does the population of released renal cells (RTC) as the source of urinary lipids vary in its cell type composition?

In lysosomal lipid storage processes of the kidney, it can be seen fine-structurally that strongly affected, slightly affected and apparently “normal” cells are mixed by various proportions. Each cell type may have a different lipid composition. The cell population released into the urine may vary statistically or accidentally in its cell type composition. Any urinary sample may have, therefore, its own lipid composition. If this effect was strong in lysosomal lipid storage processes and also in the present patient, it would impair the standardisation of single lipid values for the comparison with other urinary samples including normal controls.

S1.4.5 Comments to the results of individual lipid changes in patient 1 as compared to the normal control.

Some of the results described in section S1.3 seem to be rather variable in the two urinalyses of patient 1, and in their differences against the control. However, when regarding the different possible variables described in sections S1.4.2 to S1.4.4, some of the variability can be explained. For example, the sometimes low factors of increase of single glycosphingolipid classes in patient 1 as compared to the used normal control may be due to the possible under-estimation of the patient lipids in comparison with the normal control as explained in section S1.4.2. Moreover, the differences between some of the apparent glycosphingolipid abundances in the two urinalyses in patient 1 could at least partially be due to the much higher abundances of phospholipids including sphingomyelin in the middle as compared to the left chromatogram in the figure, for the reasons outlined in section S1.4.2 and the hypothesis in S1.4.4. The glycosphingolipid, G_M3_ ganglioside (fraction 1 in the figure), apparently has a very different abundance in the two chromatograms from patient 1. The reason is not clear, but the remark at the end of section S1.4.3 and the hypothesis in S1.4.4 may allow some understanding.

S1.5 Conclusions

Despite the problems and potential variables in the method as outlined above, the used assay of urinary lipid concentrations has revealed some findings viewed as being specific for the lysosomal disease in patient 1. When comparing in the above figure the chain of glycosphingolipid fractions numbered 2, 3, 4, 5, 6 in the two patient chromatograms (the exception of fraction 1 was explained), it can be seen that the chain has remained relatively unchanged from the first to the second urinalysis in the patient, with high lipid abundances in particular in the fractions numbered 6, and lower abundances in the fractions 4 and 2, as compared to the even lower abundances in the control chromatogram. This may support the idea outlined in section S1.4.3 that in the patient’s RTC, these glycosphingolipids may be relatively fixed within the lysosomal storage compartments and may, therefore, be less variable than the lipids outside the lysosome such as the phospholipids which have distinctly changed between the two urinalyses.

The standardisation of urinary lipids for their comparison in different urine samples is a not completely solved problem. One reason is the extreme variability in the urine composition. The used approach by expressing the individual lipid concentrations as proportions of the total lipid mass analysed has some advantages, but when comparing urinary samples with pathologic lipid increases to normal control samples, there is a tendency to over-estimating the lipids in control urines. A correction factor could come from the ratio between the absolute lipid contents in different urinary samples, but these absolute measures vary almost as highly as the urine composition.

In the absence of a fully reliable standardisation method for urinary lipid concentrations, the selective determination of one single lipid in different urinary samples would underlie the mentioned variabilities and could not exactly reflect the difference between the samples. The context of additional lipids determined in the same samples can help to decide whether a “remarkable” quantitative change of a single lipid is only the expression of all or many other lipids changed in the same way so that the risk of unspecific variability is high, or the change in fact is remarkable, which fact however, should additionally be checked in view of the other described, possible variabilities.

S1.6 References

[Iwamori M](http://www.ncbi.nlm.nih.gov/pubmed/?term=Iwamori%20M%5BAuthor%5D&cauthor=true&cauthor_uid=739008), [Nagai Y](http://www.ncbi.nlm.nih.gov/pubmed/?term=Nagai%20Y%5BAuthor%5D&cauthor=true&cauthor_uid=739008) (1978) GM3 ganglioside in various tissues of rabbit. Tissue-specific distribution of N-glycolylneuraminic acid-containing GM31. J Biochem 84:1609-1615

Tsukuda Y, Iwasaki N, Seito N, Kanayama M, Fujitani N, Shinohara Y, Kasahara Y, Onodera T, Suzuki K, Asano T, Minami A, Yamashita T (2012) [Ganglioside GM3 has an essential role in the pathogenesis and progression of rheumatoid arthritis.](http://www.ncbi.nlm.nih.gov/pubmed/22768242) PLoS One 7:e40136. doi: 10.1371/journal.pone.0040136

The other references are found in the list of the main article
